# Complete Lipooligosaccharide Structure from *Pseudoalteromonas nigrifaciens* Sq02-Rif^r^ and Study of Its Immunomodulatory Activity

**DOI:** 10.3390/md19110646

**Published:** 2021-11-19

**Authors:** Rossella Di Guida, Angela Casillo, Antonietta Stellavato, Celeste Di Meo, Soichiro Kawai, Jun Kawamoto, Takuya Ogawa, Tatsuo Kurihara, Chiara Schiraldi, Maria Michela Corsaro

**Affiliations:** 1 Department of Chemical Sciences, University of Naples “Federico II”, Complesso Universitario Monte S. Angelo, Via Cintia 4, 80126 Naples, Italy; ross.diguida@gmail.com (R.D.G.); angela.casillo@unina.it (A.C.); 2 Department of Experimental Medicine, Section of Biotechnology Medical Histology and Molecular Biology, University of Campania “Luigi Vanvitelli”, Via L. De Crecchio n 7, 80138 Naples, Italy; antonietta.stellavato@unicampania.it (A.S.); celeste.dimeo@unicampania.it (C.D.M.); chiara.schiraldi@unicampania.it (C.S.); 3 Institute for Chemical Research, Kyoto University, Kyoto 611-0011, Japan; s-kawai@nibiohn.go.jp (S.K.); jun_k@mbc.kuicr.kyoto-u.ac.jp (J.K.); ogawa.tky@mbc.kuicr.kyoto-u.ac.jp (T.O.); kurihara@scl.kyoto-u.ac.jp (T.K.)

**Keywords:** cold-adapted, endotoxin, TLR-4, LPS, structural characterization, NMR, mass spectrometry, lipid A, fish-gut bacterium

## Abstract

Lipopolysaccharides (LPS) are surface glycoconjugates embedded in the external leaflet of the outer membrane (OM) of the Gram-negative bacteria. They consist of three regions: lipid A, core oligosaccharide (OS), and O-specific polysaccharide or O-antigen. Lipid A is the glycolipid endotoxin domain that anchors the LPS molecule to the OM, and therefore, its chemical structure is crucial in the maintenance of membrane integrity in the Gram-negative bacteria. In this paper, we reported the characterization of the lipid A and OS structures from *Pseudoalteromonas nigrifaciens* Sq02-Rif^r^, which is a psychrotrophic Gram-negative bacterium isolated from the intestine of *Seriola quinqueradiata*. The immunomodulatory activity of both LPS and lipid A was also examined.

## 1. Introduction

Psychrophiles and psychrotrophs, also called cold-adapted bacteria, are microorganisms that thrive at low temperatures in permanently cold environments (0–10 °C). While psychrophilic organisms are unable to grow above 20 °C, psychrotrophs grow well at temperatures close to the freezing point of water but have their fastest growth rates above 20 °C [1].

These species have developed a wide range of adaptation strategies [1,2,3,4], such as the alteration of the membrane components to reinforce the cell envelope and to provide protection and facilitate adaptation. The structural modifications include the presence of unsaturated, short, and branched fatty acids in phospholipids that maintain membrane fluidity [4] and the different phosphorylation of membrane proteins and lipopolysaccharides (LPSs) [5,6]. LPSs are surface glycoconjugates, exposed on the external leaflet of the outer membrane (OM) of the Gram-negative bacteria, that consist of three regions named lipid A, core oligosaccharide (OS), and O-specific polysaccharide or O-antigen [7,8]. Lipid A is the glycolipid domain that anchors the LPS molecule to the OM, and therefore, its chemical structure is crucial in the maintenance of membrane integrity and the enhancement of the membrane fluidity in cold-adapted bacteria. Consistent with the observed modification in fatty acids composition, lipid A of the obligate psychrophile *Colwellia psychrerythraea* 34 H contains unsaturated acyl chains both as primary and/or secondary fatty acids [9]. The glycolipid portion is linked to the O-antigen through OS. The O-antigen is situated at the interface between the bacterium and its environment, and many of the LPSs characterized from cold-adapted bacteria lack this portion [6]. In this case, the LPS is named rough LPS or R-LPS (lipooligosaccharide, LOS) [10]. The lack of the O-specific polysaccharide could enhance the flexibility and stability of the OM.

The lipid A represents the main component of LPS from Gram-negative bacteria recognized by the immune system. It has been extensively demonstrated that the native lipid A from *Escherichia coli* activates the Toll-like receptor 4 (TLR-4) signaling, resulting in potent pro-inflammatory activity. Furthermore, the lipid A structural variation in the number or length of the acyl chain can markedly affect host immune responses. The innate immune responses are mediated by the activation of the TLR-4 and subsequent activation of the transcription factors NF-κB (nuclear factor kappa-light-chain-enhancer of activated B cells) together with the production of the pro-inflammatory cytokine interleukin 6 (IL-6) [11]. LPSs are generally able to trigger these responses that in some cases may lead to a detrimental effect, while in other cases, due to the diverse LPS structure and/or doses, they may mediate an alert to the immune system to help the tissue and organism to better face pathological attacks.

*Pseudoalteromonas nigrifaciens* Sq02-Rif^r^ is a psychrotrophic Gram-negative bacterium isolated from the intestine of a *Seriola quinqueradiata*. In this article, we report the complete structural characterization of the LOS molecule produced from *P. nigrifaciens* Sq02-Rif^r^ grown at 18 °C. Furthermore, we compared the obtained structures with those reported for *Pseudoalteromonas haloplanktis* TAC 125 [12] and *Pseudoalteromonas haloplanktis* TAB 23 [13], which are psychrophilic bacteria isolated from the Antarctic Ocean and belonging to the same genus. Finally, here, we investigate the adjuvant and immunostimulatory effects of the LPS and lipid A molecules on enterocytes obtained by the differentiation of CaCo-2 cells. Furthermore, LPS treatments were also evaluated on a gut inflammation model of Caco-2 and THP-1 cells co-culture to assess the potential immune-modulation activity [14].

## 2. Results

### 2.1. LPS Extraction

**Figure 1:**
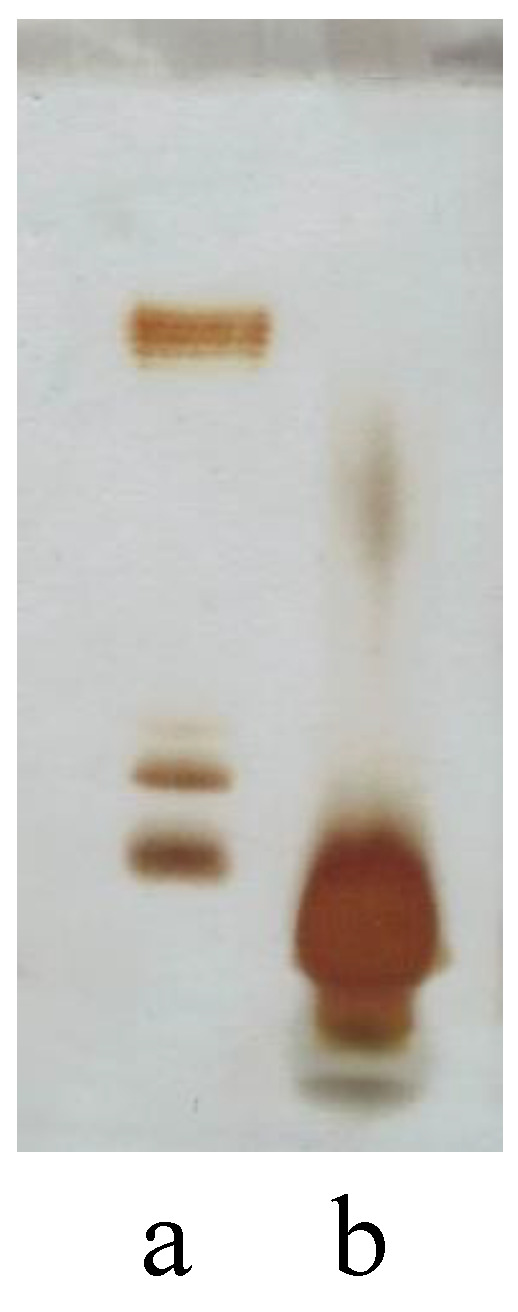
14% DOC-PAGE analysis stained with silver nitrate of (**a**) smooth-LPS from *E. coli* O55:B5 used as a standard and (**b**) PCP extract from *P. nigrifaciens* Sq02.

Dried cells of *P. nigrifaciens* Sq02-Rif^r^ (a spontaneous rifampicin-resistant mutant of *P. nigrifaciens* Sq02), grown in Luria Bertani (LB) medium at 18 °C, were extracted by the phenol/chloroform/petroleum ether (PCP) method [15]. To identify the nature of the LPS, the PCP extract was analyzed by 14% DOC-PAGE electrophoresis and visualized after silver nitrate staining. The DOC-PAGE experiment (Figure 1, lane b) showed the presence of only bands with a low molecular mass, suggesting the rough-LPS (LOS). 

### 2.2. LOS Chemical Analysis

The monosaccharide and fatty acid composition of the PCP extract was determined through their derivation as acetylated methyl glycosides (AMG) and fatty acids methyl esters (FAME), respectively. AMG and FAME were analyzed by GC-MS and identified from the retention time and fragmentation pattern by comparison with those of authentic standards. The fatty acid analysis revealed mainly the presence of 3-hydroxydodecanoic [C12:0(3-OH)] and dodecanoic (C12:0) acids. A minor amount of 3-hydroxydecanoic [C10:0(3-OH)], 3-hydroxyundecanoic [C11:0(3-OH)], 3-hydroxytridecanoic [C13:0(3-OH)], tridecenoic (C13:1), tetradecenoic (C14:1), tetradecanoic (C14:0), and pentadecanoic acids (C15:0) was also detected. Moreover, the results obtained from AMG and the octyl derivative analyses suggested the presence of D-galactose (D-Gal), D-glucosamine (D-GlcN), D-mannosamine (D-ManN), and L-*glycero*-D-*manno*-heptose (L,D-Hep). 

The AMG analysis performed after HF treatment revealed the additional presence of 3-deoxy-D-*manno*-oct-2-ulosonic acid (Kdo), suggesting its phosphorylation [13]. Finally, the GC-MS analysis of the sugars as partially methylated alditol acetates (PMAAs) revealed the following points of attachment: 3-substituted Gal (3-Gal), 4-substituted Hep (4-Hep), terminal non-reducing ManN (t-ManN), and 6-substituted GlcN (6-GlcN). All these results resembled those obtained from the LOS of the cold-adapted *Pseudoalteromonas haloplanktis* TAC 125, which was isolated from the Antarctic Ocean [12]. 

### 2.3. MALDI-TOF Analysis

**Figure 2:**
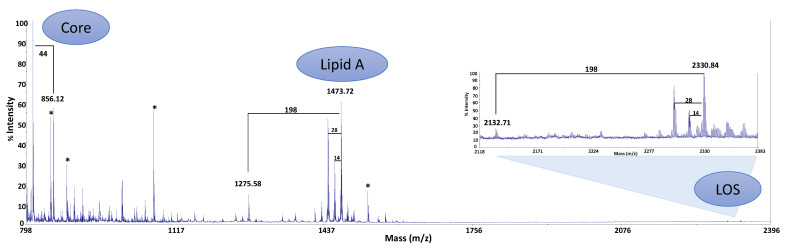
Negative ions MALDI-TOF mass spectrum recorded in reflector mode of the LOS from *P. nigrifaciens* Sq02-Rif^r^. The notation ‘14’ and ‘28’ indicate differences of one or two units of CH_2_ in the fatty acid chains, respectively. ‘198’ and ‘44’ indicate mass differences of a C12:0(3OH) and CO_2_ molecule, respectively. Asterisks “*” indicate signals not attributable to the LOS.

The intact LOS was analyzed by negative ions MALDI-TOF in reflector mode. At higher *m*/*z* values, the acquired spectrum revealed the presence of a signal at *m*/*z* 2330.84, which was attributable to a penta-acylated di-phosphorylated LOS molecule with the following composition: HexHexNAcHepKdoPGlcN_2_P_2_[C12:0(3OH)]_4_(C12:0) (calculated [M-H]^−^ = 2331.12 Da) (Figure 2). The spectrum also showed the presence of a signal at *m*/*z* 2132.71, which was attributable to a tetra-acylated species (calculated [M-H]^−^ = 2132.96 Da), which differs from the previous one in that there is the absence of a C12:0(3OH) residue (Figure 2). The same species were also observed in the ESI-MS spectrum of the LOS from *P. haloplanktis* TAC 125, although in that case, the most abundant species was the tetra-acylated [12]. 

At lower molecular masses, signals attributable to lipid A and core fragments were visible. These latter arise from an in-source breaking of the glycosidic bond between the Kdo and the lipid A. The signals at *m*/*z* 1473.72 and 1275.58 (calculated [M-H]^−^ = 1473.90 and 1275.74 Da, respectively) were attributed to lipid A fragments differing for a [C12:0(3-OH)] unit (Figure 2). Moreover, signals at *m*/*z* 856.12 and 812.13 were both assigned to the core, with a difference of 44 Da due to the loss of a CO_2_ molecule from the Kdo residue (calculated [M-H]^−^ = 856.21 and 812.22 Da, respectively).

### 2.4. Structural Characterization of the Lipid A

**Figure 3:**
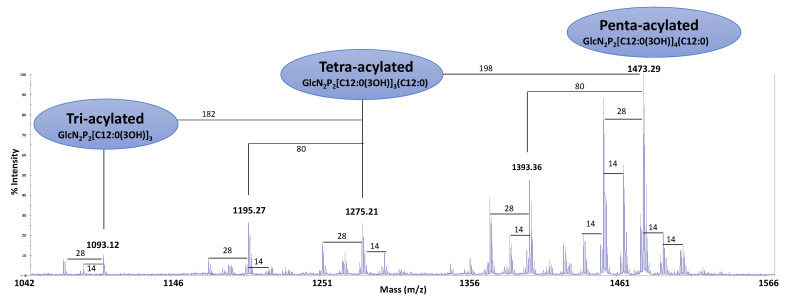
Negative ions MALDI-TOF mass spectrum recorded in reflector mode of the *P. nigrifaciens* Sq02 lipid A. The notation ‘14’ and ‘28’ indicate differences of one or two units of CH_2_ in the fatty acid chains, respectively. ‘198’, ‘182’, and ‘80’ indicate mass differences of a C12:0(3OH), a C12:0, and a phosphate group, respectively.

After treatment of the LOS with 1% acetic acid solution, the water-soluble OS was recovered in the aqueous phase and the hydrophobic lipid A moiety as a precipitate. The negative ions MALDI-TOF spectrum recorded in reflector mode of the lipid A revealed three clusters of signals, corresponding to glycoforms with different acyl substitutions (Figure 3). The signals at *m/z* 1473.29 and 1275.21 correspond to penta- and tetra-acylated di-phosphorylated glycoforms, respectively. The difference between the two signals is 198 u, corresponding to a C12:0(3OH) unit. To the signal at *m/z* 1473.29, the following composition was attributed: GlcN_2_P_2_[C12:0(3-OH)]_4_(C12:0) (Calculated [M−H]^−^ *m/z* 1473.91). Less intense are the signals assigned to di-phosphorylated tri-acylated species at *m/z* 1093.12 (Calculated [M − H]^−^ *m/z* 1093.57). The same heterogeneity of the lipid A from *P. nigrifaciens* Sq02-Rif^r^ has already been reported for other marine cold-adapted bacteria belonging to the genus *Pseudoalteromonas* [13,16,17] and for the marine *Pseudoalteromonas nigrifaciens* IAM 13010T [18].

**Figure 4:**
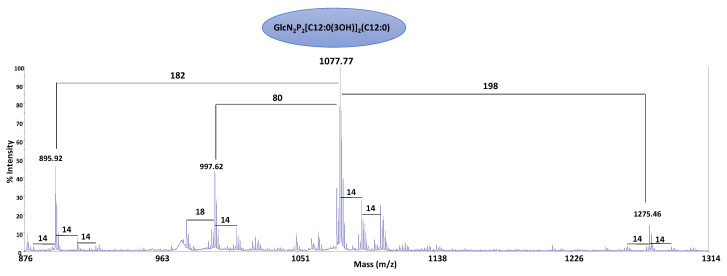
Negative ions MALDI-TOF mass spectrum recorded in reflector mode of the NH_4_OH-treated lipid A from *P. nigrifaciens* Sq02. The notation ‘14’ indicates a difference of one unit of CH_2_ in the fatty acid chains. ‘198’ and ‘182’ indicate differences of a C12:0(3OH) and C12:0, respectively.

An alkaline hydrolysis with NH_4_OH was performed to identify the position of the secondary fatty acids. This procedure leads to a lipid A devoid of acyl and acyloxacyl esters. The negative ions MALDI-TOF spectrum of the product (Figure 4) showed the presence of a signal at *m/z* 1077.77 attributed to a di-phosphorylated tri-acylated lipid A species with two C12:0(3-OH) fatty acid residues linked as amides, one of which is substituted at O-3 by a secondary C12:0 acyl chain (Calculated [M−H]^−^ *m/z* 1077.58).

To establish which of the two GlcN units carries the C12:0 as secondary fatty acid, a tandem mass experiment was performed on the intact lipid A sample. The negative ions MS/MS spectrum obtained from the precursor ion at *m/z* 1473.29 revealed the presence of several signals (Table 1). Such peaks were attributable either to the loss of a phosphate group and acyl substituents or to inter-residue fragmentations. In particular, Y_1_ and B_1_ fragments [19], at *m/z* 654.65 and 818.88, respectively, suggested that the non-reducing GlcN unit bears the C12:0. 

Therefore, all the above MS experiments indicated that the main species of the lipid A of the LOS isolated from *P. nigrifaciens* Sq02-Rif^r^ is a pentacylated glycoform with the position of the secondary fatty acid as acyloxamide on the non-reducing glucosamine residue. This distribution of fatty acids is identical to that found for lipid A of the cold-adapted bacterium *Pseudoalteromonas haloplanktis* TAB 23 [13].

### 2.5. LOS De-Acylation and NMR Analysis

**Figure 5:**
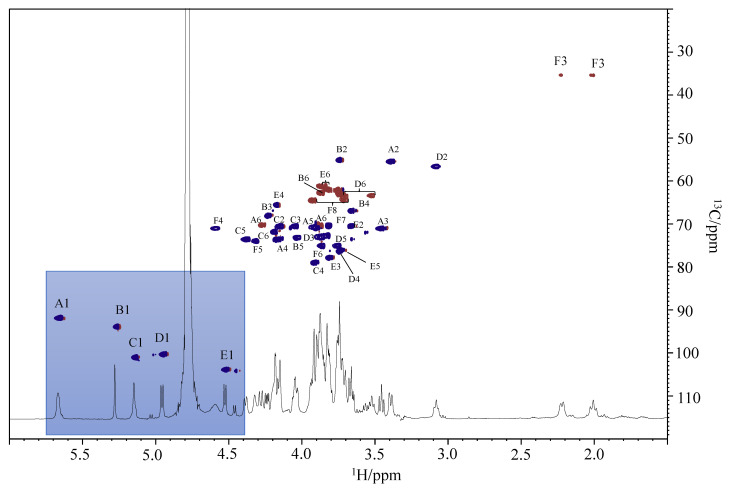
^1^H and ^1^H,^13^C DEPT-HSQC spectra of the oligosaccharide from the LOS of *P. nigrifaciens* Sq02-Rif^r^. The spectra were recorded at 298 K in D_2_O at 600 MHz.

To confirm the structure of its oligosaccharide moiety, the LOS was fully de-acylated. The LOS sample was de-O-acylated by mild hydrazinolysis and then de-N-acylated by strong alkaline hydrolysis with 4 M of KOH. The resulting mixture was desalted on a Sephadex column, and the obtained oligosaccharide fraction was analyzed by NMR experiments. All the ^1^H and ^13^C chemical shifts (Table 2) of oligosaccharide were obtained by 2D NMR spectroscopic experiments (^1^H-^1^H COSY, ^1^H-^1^H TOCSY, ^1^H-^1^H ROESY, ^1^H-^13^C DEPT-HSQC, ^1^H-^13^C HMBC). The ^1^H-^13^C HSQC spectrum (Figure 5), showed in the anomeric region five signals, correlated with anomeric carbon, and at low chemical shifts two signals (2.23 and 2.01 ppm, respectively) diagnostic of the axial and equatorial diastereotopic H-3 protons of the Kdo. 

The anomeric configurations were deduced from the ^1^*J*_C_,_H_ coupling constant values measured in a 2D F*2*-coupled HSQC experiment. The first three residues (**A**–**C**) were identified as α-configured (185, 176, and 179 Hz, respectively), while **D** and **E** were β-configured (171 and 165 Hz, respectively).

Residue **A** (with H-1/C-1 at δ 5.65/91.9 ppm) was identified as the proximal GlcN unit of the lipid A, which is based on the multiplicity of the anomeric proton signal due to its phosphorylation and on the C-2 chemical shift at δ 55.5 ppm, indicating a nitrogen-bearing carbon atom. Moreover, the C-6 linkage position of this residue was suggested from the glycosylation shift of its C-6 value at δ 70.3 ppm. The residue **D**, with H-1/C-1 signals at δ 4.94/100.4 ppm, was assigned to the distal β-GlcN4P of the lipid A because of its C-2 chemical shift at δ 56.7 ppm and the long-range scalar coupling between H-1 of **D** and C-6 of **A** in the HMBC spectrum. Moreover, the downfield shifts of H-4/C-4 is diagnostic for the presence of a phosphate group linked at O-4 [20]. The β-anomeric configuration of this residue was confirmed by the intra-residue NOE correlations observed between 1-H and both H-3 and H-5 in the ROESY spectrum. 

Residue **B**, with H-1/C-1 at δ 5.26/93.9 ppm, was recognized as *manno*-configured, since in the TOCSY experiment, the H-1 signal showed a correlation only with H-2. In addition, H-2 is correlated with a carbon linked to a nitrogen atom at δ 55.2 ppm. This residue was identified as a terminal mannosamine (t-ManN) since no glycosylation shift of its carbons was observed [21]. 

The residue **C**, with H-1/C-1 at 5.12/101.1 ppm, was identified as a heptose due to the presence in the TOCSY spectrum only of the correlation between H1 and H2, which suggests a *manno*-configuration. Residue **C** was found to be 4-substituted, since the chemical shift of its C-4 was downshifted compared to the reference value for an unsubstituted residue [13].

Residue **E** was attributed to a *galacto*-configured residue based on the correlation in the TOCSY experiment of its anomeric proton with only four protons. The β anomeric configuration of E was suggested by the intra-residue NOE correlations between H-1/H-3 and H-1/H-5, and by the value of ^1^*J*_C,H_ 165 Hz. Due to the downfield shift of its C-3, with respect to that of an unsubstituted galactose residue [21], residue **E** was assigned to a 3-substitued galactose.

Residue **F** was identified as a Kdo unit starting from the two diastereotopic protons of the C-3 at δ 2.23 and 2.01 ppm. In the TOCSY experiment, these two protons were correlated to signals at δ 4.59 and 4.31 ppm, which are assigned to H-4 and H-5, respectively. The downfield shift of the C-5 carbon signal, compared to the value for the unsubstituted monosaccharide [22], at 74.0 ppm is an index of its glycosylation at the O-5 position. Moreover, the H-4 and C-4 chemical shifts, downfield shifted at δ 4.59 and 71.7 ppm, respectively, indicated the Kdo phosphorylation at this position [23]. Finally, the α-configuration of this residue was suggested by the difference (Δ = 0.22) between H-3_ax_ and H-3_eq_ chemical shifts [24].

**Figure 6:**
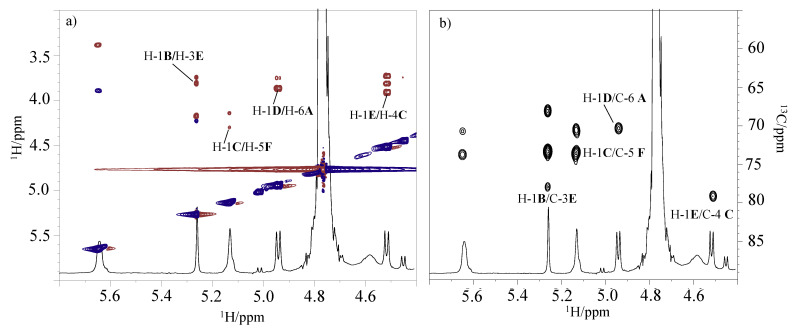
(**a**) Inter-residue correlations of ROESY spectrum and (**b**) long-range scalar connectivities of HMBC spectrum of the oligosaccharide from the LOS of *P. nigrifaciens* Sq02-Rif^r^. The spectra were recorded in D_2_O at 298 K at 600 MHz.

The sequence of the monosaccharide residues was deduced by using NOE inter-residues correlations and long-range scalar connectivities (Figure 6). In particular, the ROESY experiment revealed inter-residues contacts assigned to H-1 of **B** with H-3 of **E**, H-1 of **E**, and H-4 of **C**, H-1 of **C** with H-5 of **F**, and H-1 of **D** with H-6 of **A**, respectively (Figure 6a). Moreover, the HMBC spectrum showed the following correlations: H-1 of **B** with C-3 of **E**, H-1 of **E** with C-4 of **C**; H-1 of **C** with C-5 of **F**, and H-1 of **D** with C-6 of **A** (Figure 6b). In addition, the C-2 of the Kdo at δ 100.7 ppm showed a long-range correlation with 6-H of residue **D** at δ 3.52 ppm, confirming its linkage to the lipid A backbone. These data agreed with the glycosylation pattern obtained from the ^13^C chemical shift of the DEPT-HSQC experiment and the linkage position of the partially methylated alditol acetates analysis. 

**Figure 7:**
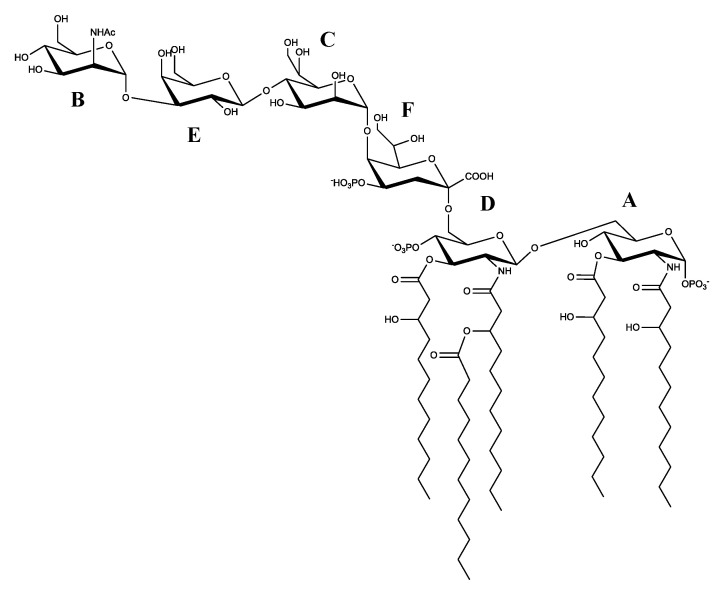
Structure of the LOS from *Pseudoalteromonas nigrifaciens* Sq02-Rif^r^.

Therefore, the LOS of *P. nigrifaciens* Sq02 Rif^r^ has the following structure (Figure 7):

### 2.6. TLR-4 Activation and IL-6 mRNA Expression Evaluated by qRT-PCR

**Figure 8:**
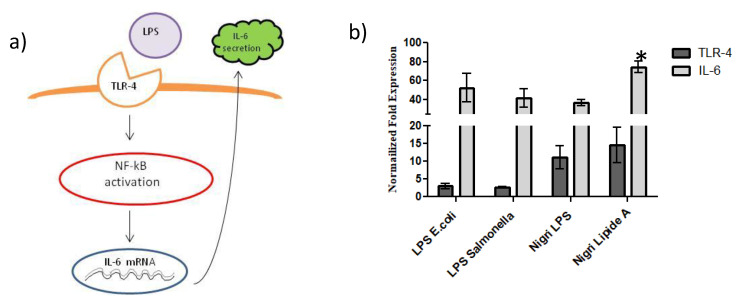
(**a**) Schematic diagram illustrating the cascade and signaling pathway activated after LPS/TLR-4 interaction. (**b**) Gene expression analyses: the results are expressed as fold change of LPS-treated cells respect to untreated cells (CTR) for TLR-4 and IL-6 in differentiated CaCo-2 cells. Data showed as the average ± SD. * *p* < 0.01 vs. CTR and vs. commercial LPS ones.

To determine the activity of LPS and lipid A fractions on the inflammatory cascade (Figure 8a), we first analyzed the transcriptional level of TLR-4 and IL-6 mRNA expression in differentiated CaCo-2 cells. In this respect, both TLR-4 and IL-6 were upregulated both in the presence of the commercial LPSs from *E. coli* O55:B5 and *Salmonella minnesota* and in the presence of the LPS and lipid A from *P. nigrifaciens*. The results reported in Figure 8 indicated that both commercial LPSs increased TLR-4 expression by about two-fold vs. control, while the LPS and lipid A isolated from *P. nigrifaciens* Sq02-Rif^r^ increased the TLR-4 expression by about 10 and 15-fold, respectively, with respect to the untreated cells (CTR) (Figure 8b). Subsequently, an increase in IL-6 mRNA expression was shown. In fact, as expected considering the TLR-4 activation, we found a significant upregulation of IL-6 for all the samples analyzed at a concentration of 20 μg mL^−1^. 

Specifically, both for commercial LPSs and *P. nigrifaciens* LPS, IL-6 expression was about 30–45-fold higher than the control (Figure 8b).

Interestingly, *P.* *nigrifaciens* Sq02-Rif^r^ lipid A increased IL-6 expression by about 80-fold vs. control. In this respect, Arbizu et al. [25] reported an increased expression of TLR-4 mRNA of about 2.5-fold with respect to control in HT-29 colon cancer cells treated with LPS at 4 µg mL^−1^ concentration [25]. This difference of gene expression compared with our data may be ascribed to the different concentrations of LPSs tested.

### 2.7. NF-κB Upregulates Protein Expression in Response to TLR-4 Activation

**Figure 9:**
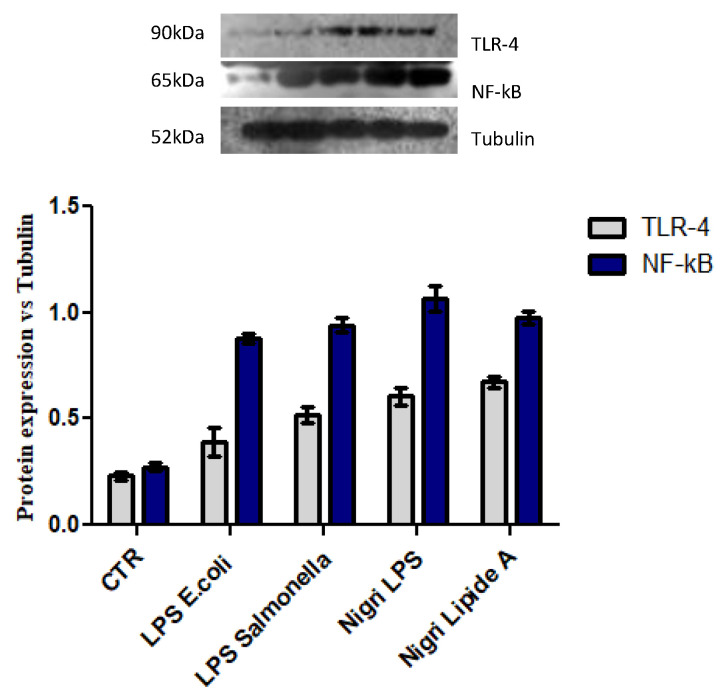
Western blotting for expression of TLR-4 and NF-kB protein signal normalized to tubulin in the densitometry reported as average and SD.

To identify and confirm the underlying signaling pathways, TLR-4 and NF-*κ*B activation were evaluated by Western blotting experiments. As shown in Figure 9, TLR-4 increase was confirmed, and all the LPSs and lipid A fractions caused higher expression than controls. Consistent with the increase in TLR-4, the expression level of NF-*κ*B also increased when compared to the control. 

### 2.8. QUANTI-THP-1 Blue Assay 

In this study, we aimed also to employ a recently available tool for the detection of NF-kB activation in monocytes to quickly highlight the activation of the inflammation pathway (through THP1-Blue™ cells). Co-cultivation of CaCo-2/THP1-Blue™ NF-κB reporter cells permitted comparison of the inflammatory modulation via the NF-*κ*B pathway between LPS- and lipid A-treated cells and untreated cells. 

**Figure 10:**
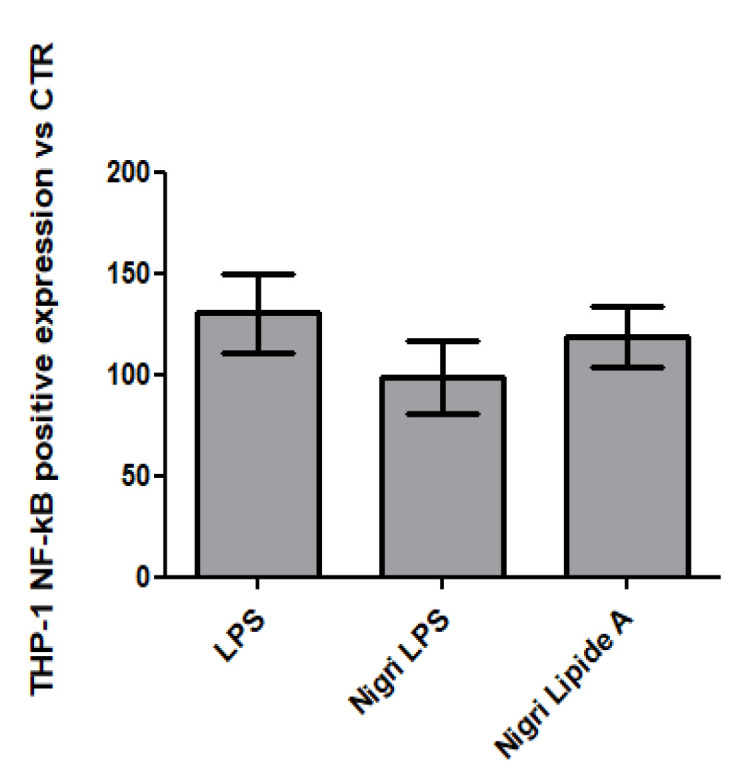
Spectrophotometric quantification of NF-*κ*B by QUANTI-Blue™ assay in cells that were untreated (CTR) or exposed to commercial LPS from Salmonella and LPS and lipid A fractions isolated from *P. nigrifaciens* Sq02-Rif^r^.

As shown in Figure 10, LPS isolated from *P. nigrifaciens* showed a similar upregulation with respect to the LPS from *Salmonella* quantified by spectrophotometric analyses, which is slightly but not significantly lower.

## 3. Materials and Methods

### 3.1. Cells Growth 

*P. nigrifaciens* Sq02-Rif^r^ was grown in 5 mL of LB medium supplemented with NaCl (LB-NaCl: 1% Bacto Tryptone (BD Difco, Detroit, MI, USA), 0.5% yeast extract (BD Difco), and 3% NaCl) containing 50 µg mL^−1^ rifampicin at 18 °C to the early stationary phase (OD_600_ = 1.0–1.5) and transferred to 1 L of LB-NaCl. The cells grown to the stationary phase (OD_600_ = 2.0–2.5) at 18 °C were harvested by centrifugation and suspended in 6 mL of Dulbecco’s phosphate-buffered saline (DPBS: 0.2 g KCl, 0.2 g KH_2_PO_4_, 11.7 g NaCl, 1.2 g Na_2_HPO_4_, 0.1 g MgCl_2_·6H_2_O, 0.1 g CaCl_2_ in 1 L) [26]. The cell suspension was centrifuged at 6800× *g* and 4 °C for 10 min, and the pelleted cells were washed twice with 4 mL of DPBS. The washed cells were lyophilized and used for LPS extraction.

### 3.2. LPS Isolation and Purification

The LOS (36.7 mg) was extracted from dried cells (4.78 g) by the PCP method [15]. The sample was washed three times with a mixture of chloroform−methanol (1:2, *v*/*v*) to remove traces of phospholipids. To evaluate the protein concentration, the Bradford method was performed (Bio-Rad Laboratories, Milano, Italy).

### 3.3. DOC-PAGE Analysis

Polyacrylamide gel electrophoresis (PAGE) was performed using the system of Laemmli [27] with sodium deoxycholate (DOC) as detergent as already reported [28]. LOS bands were visualized after silver nitrate staining [29]. 

### 3.4. Chemical Analysis

The monosaccharides were analyzed as acetylated methyl glycosides by GC-MS [28]. Briefly, an aliquot of LPS sample (0.5 mg) was subjected to a methanolysis reaction with HCl/CH_3_OH (1.25 M, 1 mL) at 80 °C for 16 h. The methanol was extracted three times with hexane to separate the fatty acid methyl esters from the O-methyl glycosides. The hexanic phase was dried and analyzed by GC-MS. The methanolic phase was dried, and then, the methyl glycosides were acetylated with acetic anhydride in pyridine at 100 °C for 30 min. The absolute configuration of the sugars was determined by subjecting the acetylated (*S*)-2-octyl glycosides to GC-MS [30]. The samples were analyzed on an Agilent Technologies gas chromatograph 7820A equipped with a mass selective detector 5977B and an HP-5 capillary column (Agilent, 30 m × 0.25 mm i.d.; flow rate, 1 mL min^−1^, He as carrier gas).

Acetylated methyl glycosides were analyzed using the following temperature program: 140 °C for 3 min, then 140→240 °C at 3 °C min^−1^. The temperature program for the analysis of acetylated octyl glycosides: 150 °C for 5 min, 150→240 °C at 6 °C min^−1^, 240 °C for 5 min. 

### 3.5. Deacylation of LOS 

The LOS sample (20.2 mg) was dried over phosphorus anhydride in a vacuum chamber and then treated with hydrazine (1.5 mL) at 37 °C for 2 h. The de-*O*-acylated LOS was precipitated by adding cold acetone. The pellet was recovered after centrifugation at 4 °C and 6300 rcf for 30 min, washed three times with acetone, dissolved in water, and lyophilized [31]. The de-*O*-acylated LOS (7.8 mg) was submitted to a reaction with 4 M KOH (1.0 mL) for 16 h at 120 °C. The reaction mixture was neutralized with 2 M HCl (until pH 6) and extracted with CHCl_3_ three times. The aqueous layer was desalted on a Sephadex G-10 column (GE Healthcare, Pittsburgh, PA, USA, 2.5 × 43 cm, 31 mL h^−1^, fraction volume 2.5 mL, eluent H_2_O). Then, the eluted fraction was lyophilized (1.4 mg).

### 3.6. De-O-Acylation of Lipid A

Lipid A (0.2 mg) sample was treated with an ammonium hydroxide solution (200 μL) for 16 h at room temperature [32]. Then, the product was dried under air flow, dissolved in water, and lyophilized.

### 3.7. Mass Spectrometry Analysis

MALDI-TOF mass spectra were acquired on an ABSCIEX TOF/TOF™ 5800 (AB SCIEX, Darmstadt, Germany) mass spectrometer. A solution of 2,4,6-trihydroxyacetophenone (THAP) in water/methanol 1:1 (10 mg mL^−1^) was used as the matrix. The LOS and lipid A samples were desalted on a Dowex 50WX8 (H^+^ form) and dissolved in 2-propanol/water with a 1:1 ratio and in chloroform/methanol 4:1, respectively. The acquired mass spectra were calibrated and processed under computer control by using the Data Explorer software.

### 3.8. NMR Spectroscopy

^1^H and ^13^C NMR mono- and two-dimensional spectra were recorded in D_2_O at 298 K with a Bruker Avance 600 MHz equipped with a cryoprobe. ^13^C and ^1^H chemical shifts were determined by using acetone as external standard (δ_H_ 2.225 and δ_C_ 31.45, respectively). All 2D homo- and heteronuclear experiments (^1^H,^1^H COSY, ^1^H,^1^H TOCSY, ^1^H,^1^H ROESY, ^1^H,^13^C-HSQC, 2D F*2*-coupled ^1^H,^13^C HSQC and ^1^H,^13^C HMBC) were performed as already reported [33]. 

### 3.9. Cell Culture

Caco-2 cells (Human Caucasian colon adenocarcinoma cells, ATCC^®^ HTB-37™) were grown in Dulbecco’s modified eagle medium (DMEM) containing glucose and glutamine and supplemented with 10% (*v*/*v*) fetal bovine serum (FBS) heat inactivated (56 °C for 30 min).

The cells were grown in a sterile 25 cm^2^ flask at a concentration of 3 × 10^5^ to confluence for 21 days to reach full differentiation in normal enterocytes [34]. THP1-Blue™ NF-κB reporter cells were obtained from In Vivo Gen (Toulouse, France) and maintained in Roswell Park Memorial Institute (RPMI) 1640 medium containing glucose (2 g L^−1^) and glutamine (0.3 g L^−1^), supplemented with 10% (*v*/*v*) FBS, 100 μg mL^−1^, Normocin™, and Pen-Strep (100 U mL^−1^). Cells were incubated at 37 °C in a humidified atmosphere of air/CO_2_ (95:5, *v*/*v*).

### 3.10. TLR-4 and IL-6 mRNA Analyses Using qRT-PCR Analyses 

TRIzol RNA Isolation Reagents (Thermo Fisher Scientific, Waltham, MA, USA) was used to isolate total cellular RNA. cDNA was reverse transcribed by Reverse Transcription System Kit (Promega, Milan, Italy) according to the manufacturer’s instructions. Quantitative real-time polymerase chain reactions (qRT-PCR) were performed in duplicate for all genes of interest using IQ ™ SYBR^®^ Green Supermix (Bio-Rad Laboratories, Milan, Italy) and internal control the glyceraldehyde-3-phosphate dehydrogenase (GAPDH) housekeeping gene. The primers used to amplify each PCR product are reported in Table 3. Values are expressed as a fold change (2^−∆∆Ct^ method) in treated cells vs. untreated cells (the control) and normalized to transcript levels of housekeeping gene [35]. 

### 3.11. Western Blotting for TLR-4, NF-κB, and Tubulin

After 48 h of treatment, Western blotting analyses were performed. Cells were lysed in Radio-Immunoprecipitation Assay (RIPA buffer; Cell Signaling Technology), and intracellular protein concentration was quantified using the Bradford method. Specifically, 50 µg of proteins for each sample were resolved on a 10% SDS–PAGE gel and transferred onto nitrocellulose membrane (GE, Amersham, UK). Then, the membrane was blocked by 5% *w*/*v* non-fat milk in Tris-buffered saline and 0.05% *v*/*v* Tween-20 (TTBS) for 30 min and incubated overnight at 4 °C with primary antibodies against TLR-4 (Abcam, Cambridge, UK) and NF-κB (Santa Cruz, Dallas, TX, USA) diluted 1:500. Then, the membrane was incubated with secondary antibodies, horseradish peroxidase-conjugated donkey anti-rabbit and goat anti-mouse, which was diluted 1:5000 for 2 h at room temperature. Anti-β-tubulin antibody diluted 1:1000 was used as the loading control. The signal was detected using the ECL system (Chemicon-Millipore, Milano, Italy), and the semi-quantitative analyses of protein expression were carried out with the ImageJ program.

### 3.12. QUANTI-THP1-Blue Assay, Caco-2/THP-1 Co-Culture

Caco-2 monolayers were cultured to confluence (triplicates for each condition) for 21 days to reach full differentiation according to previously reported protocols [34]. In order to obtain a gut inflammation model, these monolayers were incubated with commercial LPS (LPS from *Salmonella minnesota* S-form, Enzo Life Sciences, Farmingdale, NY, USA) and LPS isolated from *P. nigrifaciens* at 20 µg mL^−1^ for 24 h. Successively, THP1-Blue™ cells were added to start the co-culture. Specifically, monocytes (THP-1) were added to the LPS-treated monolayers at a concentration of 5 × 10^4^ cells mL^−1^ to obtain a co-culture model [14,36]. Supernatants were collected after 6 h incubation for NF-κB quantification (QUANTI-Blue reagent, InvivoGen, Toulouse, France). The assay was performed following the manufacturer’s instructions, and luminescence intensity was quantified at 655 nm using a Microplate Reader (Biorad Laboratories, Milan, Italy) to determine SEAP (secreted embryonic alkaline phosphatase) levels.

## 4. Conclusions

The structure of the lipooligosaccharide from the psychrotrophic Gram-negative bacterium *Pseudoalteromonas nigrifaciens* Sq02-Rif^r^, isolated from the intestine of a *Seriola quinqueradiata* (yellowtail), was determined by chemical analysis, NMR spectroscopy, and MALDI-TOF mass spectrometry. In this study, we demonstrated that the LOS isolated from *P. nigrifaciens* Sq02-Rif^r^ shares the same core oligosaccharide structure with *P. haloplanktis* TAC125 [12]. Confirmation of this result was achieved by studying the 2D NMR spectra of the fully deacylated LOS (OS).

Moreover, lipid A displays the same fatty acid composition and heterogeneity already reported for other marine cold-adapted *Pseudoalteromonas* species [13,16,17] and for *P. nigrifaciens* IAM 13010^T^ [18]. These structures consist of a di-phosphorylated disaccharide GlcN backbone carrying four C12:0(3-OH) and a C12:0 residue and differ regarding the position of the secondary fatty acid. The MS analysis of the lipid A from *P. nigrifaciens* Sq02-Rif^r^ revealed that the C12:0 is the substituent of the N-linked primary fatty acid of the non-reducing GlcN, as already found for *P. haloplanktis* TAB 23 and *P. nigrifaciens* IAM 13010^T^.

Finally, the immunomodulatory effect of the LPS and the lipid A was studied showing similarities to the commercial LPS from *E. coli* and *S. minnesota*. In fact, differentiated CaCo-2 cells increased TLR-4 expression at mRNA and protein levels following LPS and lipid A treatments. Then, the NF-κB pathway was activated toward cytokines production [11,37,38]. In this respect, the THP-1 blue NF-κB reporter assay gave a further indication of the induction inflammation cascade in response to the LPSs/lipid A binding, further validating the RT-PCR results.

It is important to underline that although lipid A from *P. nigrifaciens* and *P. haloplanktis* TAB 23 share the same primary structure, they exhibit a different immunostimulant effect on the treated cells. While *P. nigrifaciens* lipid A shows a promising stimulant activity on enterocytes cells, *P. haloplanktis* TAB 23 does not provide the same effect on the THP-1 cell line, suggesting that not only the acyl distribution but also the cell line used as the target can influence the biological activity.

## Figures and Tables

**Table 1:** Main fragment signals observed in the negative ions MALDI-TOF MS/MS spectrum, acquired in reflector mode, of the precursor ion [M-H]^−^ at *m/z* 1473.29.

1258.38	[M-H]^−^-C12:0(3-OH)-H_2_O
1178.34	[M-H]^−^-C12:0(3-OH)-H_2_O-P
980.01	[M-H]^−^-[C12:0(3-OH)]_2_-H_2_O-P
818.88	B_1_
654.65	Y_1_

**Table 2:** ^1^H and ^13^C NMR assignments of the oligosaccharide from the LOS of *P. nigrifaciens* Sq02-Rif^r^ grown at 18 °C. Spectra were recorded in D_2_O at 298 K at 600 MHz, using acetone as external standard (δ_H_/δ_C_ 2.225/31.45 ppm).

Sugar Residue	H1 C1 ^1^*J*_C_,_H_	H2 C2	H3 C3	H4 C4	H5 C5	H6 C6	H7 C7	H8 C8
**A** α-6-d-GlcN*p*1P	5.65 91.9 185 Hz	3.39 55.5	3.45 71.1	4.16 73.6	3.89 70.9	4.26, 387 70.3		
**B** α-t-d-Man*p*N	5.26 93.9 176 Hz	3.74 55.2	4.23 68.2	3.65 66.9	4.03 73.2	3.86 62.8		
**C** α-4- L,d-Hep*p*	5.12 101.1 179 Hz	4.14 70.6	4.04 70.5	3.91 78.9	4.37 73.6	4.19 71.9	3.74 62.2	
**D** β-6-d-GlcN*p*4P	4.94 100.4 171 Hz	3.08 56.7	3.86 72.9	3.74 76.5	3.75 75.1	3.52, 3.71 63.4		
**E** β-3-d-Gal*p*	4.51 103.9 165 Hz	3.66 70.5	3.80 77.9	4.16 65.6	3.73 76.2	3.83, 3.87 61.3		
**F** α-5-d-Kdo*p*4P	- 175.7	- 100.7	2.23, 2.01 35.5	4.59 71.7	4.31 74.0	3.86 75.0	3.81 70.5	3.92, 3.71 64.6

**Table 3:** Oligonucleotide sequences used in this study.

Gene Name (Symbol)	PCR Primer Sequence 5′→ 3′	Annealing Temperature (°C)
Glyceraldehyde-3-phosphate dehydrogenase (GAPDH)	TGCACCACCAACTGCTTAGC GGCATGGACTGTGGTCATGAG	55
Toll like receptor 4 (TLR-4)	TCCCAggAATTggTgATAAAgTAgA CTggCATgACgCgAACAATA	55
Interleukin 6 (IL-6)	GTGGAGATTGTTGCCATCAACG CAGTGGATGCAGGGATGATGTTCTG	55
